# Simultaneously Enhanced Potential Gradient and Nonlinearity of ZnO Varistor Ceramics by MnO Doping with Nano-Sized ZnO Powders

**DOI:** 10.3390/ma14247748

**Published:** 2021-12-15

**Authors:** Yao Wang, Zongke Hou, Jianying Li, Kangning Wu, Jiguang Song, Rui Chen, Kai Li, Liucheng Hao, Chenbo Xu

**Affiliations:** 1 State Key Laboratory of Electrical Insulation and Power Equipment, Xi’an Jiaotong University, Xi’an 710049, China; w3119304391@stu.xjtu.edu.cn (Y.W.); houzongke@stu.xjtu.edu.cn (Z.H.); 2 Pinggao Group Co., Ltd., Pingdingshan 467001, China; song651700317@126.com (J.S.); chenrui1009@126.com (R.C.); 007zzulikai@163.com (K.L.); 13781871591@163.com (L.H.); 3 State Grid Zhejiang, Electric Power Co., Ltd., Hangzhou 310000, China; zjlsxcb@163.com

**Keywords:** ZnO, varistor ceramics, high potential gradient, nonlinearity, Schottky barrier

## Abstract

ZnO varistor ceramics with a high potential gradient, as well as a high nonlinear coefficient, were reported and analyzed in this paper. With the use of nano-sized ZnO powders, the average grain size was reduced to about 2.6 μm, which successfully raised the potential gradient to 1172 V/mm. Moreover, the nonlinear coefficient increased to 48, and the leakage current was decreased to 8.4 μA/cm^2^ by doping a moderate amount of MnO (0.9 mol%). This was proven to be caused by the high Schottky barrier height formed at the grain boundary, where the Mn element segregated and, consequently, led to the increased density of interface states. Therefore, this could be considered as a potential method to simultaneously enhance the potential gradient and the nonlinear coefficient of ZnO varistor ceramics.

## 1. Introduction

ZnO varistor ceramics are widely employed to protect electrical power systems and electronic devices from surges and overvoltage due to their excellent nonlinear current–voltage characteristics and energy absorption capability [1,2]. High-performance ZnO varistor ceramics with a high potential gradient, high nonlinear coefficient and low leakage current are urgently required due to the increasing requirements in the fields of device miniaturization, ultra-high-voltage power systems, high-speed electrified railways, and integrated circuits, etc. [3,4,5,6,7]

The potential gradient (*E*_1mA_) of ZnO varistor ceramics is proportional to the number of grain boundaries (GB) per unit length and the breakdown voltage of a single GB, which is approximately constant at ~3 V [1]. Therefore, the commonly accepted approach to enhance the potential gradient is to reduce the grain size, which is generally achieved by optimizing doping [4,8,9,10], sintering [11,12,13] and nano-sized raw materials [5,14,15,16,17,18,19]. Among these methods, the most effective method is using nano-sized ZnO powders to prepare ZnO varistor ceramics. It was reported that the high *E*_1mA_ of 800 V/mm was achieved by using nano powers with nonlinear coefficients (α) of approximately 20 [5,20]. Similarly, ZnO varistors, with a high α of 96, were prepared but followed by the high leakage current density (*J*_L_) of 50 μA/cm^2^ [21]. This is because a small grain size would increase the number of grain boundaries which would weaken the Schottky barrier and lower its barrier height, resulting in a low α and high *J*_L_. Therefore, it seems hard to co-achieve a high potential gradient, high nonlinearity and low leakage current for ZnO varistor ceramics [22,23,24,25]. When the high potential gradient is more than 1000 V/mm, it is difficult for the nonlinear coefficient to reach more than 40, and, at the same time, the leakage current density is seldom lower than 10 μA/cm^2^ [5,20,26,27,28,29]. The electrical properties of ZnO varistor ceramics are expected to largely depend on their composition. The doping of a transition metal element, e.g., Mn, is acknowledged as a feasible method to improve electrical nonlinearity and reduce the leakage current of ZnO varistor ceramics. Of course, there are many studies on the preparation of ZnO varistor ceramics by doping MnO with micron ZnO powders [20,30,31,32,33]. However, the effects of MnO doping (0.3–1.2 mol%) on the electrical properties of ZnO varistor ceramics prepared by nano sized ZnO powers are inconclusive.

In this paper, ZnO varistor ceramics with a high potential gradient of 1172 V/mm, high nonlinear coefficient of 48 and low leakage current of 8.4 μA/cm^2^, were obtained by doping MnO with nano-sized ZnO powers. The effects of MnO content on the microstructures, Schottky barriers and electrical properties of ZnO varistor ceramics were investigated. This could provide helpful references for preparing high-performance ZnO varistor ceramics.

## 2. Experiments

A series of ZnO varistor ceramic samples were prepared through the solid-state reaction method with the following raw materials: (96.2-*x*) mol% ZnO, 1.3 mol% Bi_2_O_3_, 1.5 mol% Sb_2_O_3_, 0.6 mol% Co_3_O_4_, 0.4 mol% Ni_2_O_3_, 0.008 mol% Al(NO_3_)_3_·9H_2_O and *x* mol% MnO (*x* = 0.3, 0.6, 0.9, 1.2). The chemical purity of the raw materials was 99.9%, 99.9%, 99.5%, 99.9%, 99%, 99.9% and 99.5%, respectively. Among these materials, the nano-sized ZnO powders (30 ± 10 nm) were used. The raw materials were milled in polyethylene bottles for 12 h. Then, the mixed slurry was dried at 80 °C. After dying, a large amount of 3 wt% polyvinyl alcohol (PVA) was added into the powders. Then, the slurry was mixed and granulated. The powders were pressed into pellets and pre-sintered at 600 °C. Then, the pellets were sintered at 1050 °C for 2 h with a heating rate of 3.33 °C/min and naturally cooled to room temperature in the air atmosphere. Finally, ZnO varistor ceramic samples with a diameter of about 11.5 mm and a thickness of about 2.5 mm were obtained. They were designated as M1, M2, M3, and M4, with the increasing MnO content.

The surface morphology, element analysis and crystal structure of samples were characterized by scanning electron microscope (SEM, VE-8600S, Keyence, Osaka, Japan), EDS (JSM6390A, JEOL, Tokyo, Japan) and X-ray diffraction (XRD, D8 Advance, Bruker, Berlin, Germany), respectively. The densities of the samples were measured by the Archimedes method. The nonlinear current density-electrical field (*J-E*) curve was measured at room temperature by using a Keithley DMM 7510 digital multi-meter and a TD2200 precision linear high-voltage DC power. The voltage dependence of barrier capacitance was measured by an impedance analyser (Novocontrol Concept 80, Frankfurt, Germany).

## 3. Results

**Figure 1:**
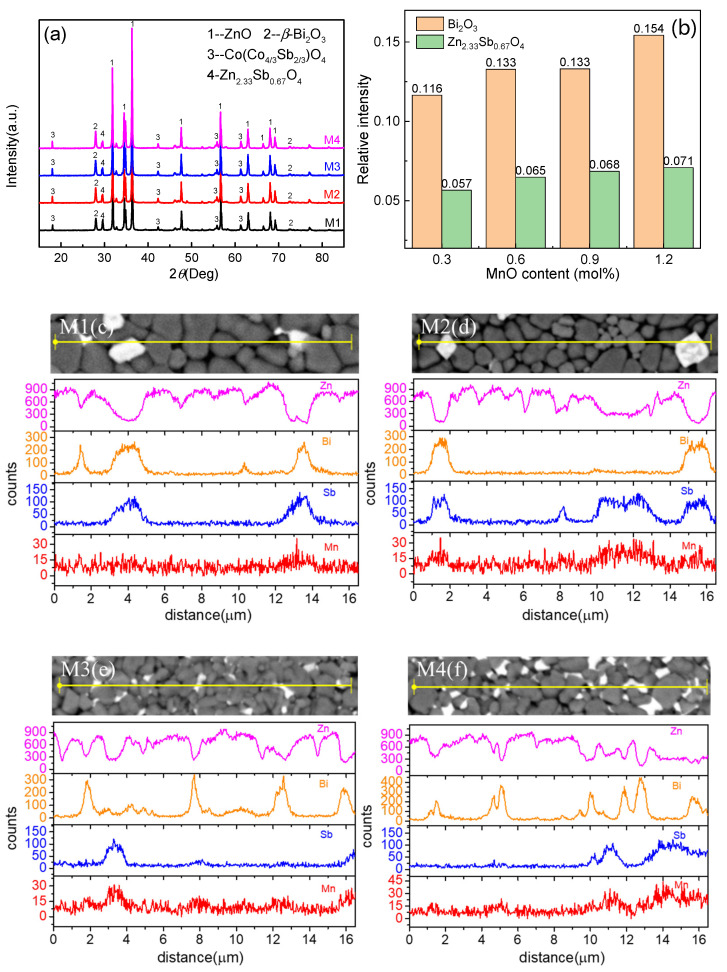
(**a**) XRD patterns, (**b**) relative intensity of Bi_2_O_3_ and (**c**–**f**) Line-scanning EDS results covering several grains and grain boundaries of ZnO varistor samples.

Figure 1a shows the XRD patterns of the ZnO varistor ceramics. The ZnO crystal phase (JCPDS Card No. 89-0510), Bi-rich phase and spinel phase Co(Co_4/3_Sb_2/3_)O_4_ (JCPDS Card No. 78-0718), and Zn_2.33_Sb_0.67_O_4_ (JCPDS Card No. 15-0687) were identified in all of the samples. The *β*-Bi_2_O_3_ phase (JCPDS Card No. 27-0050) and spinel phase were the same in different samples. Those results indicated that the MnO doping content had little effect on the crystal phase of ZnO varistor ceramics, which was in accordance with other reports [20]. In order to compare the content of the intergranular phase of different samples, the Bi_2_O_3_ phase and Zn_2.33_Sb_0.67_O_4_ are taken as examples. The ratio of peak height of the Bi_2_O_3_ phase and Zn_2.33_Sb_0.67_O_4_ to that of the ZnO main crystal phase in different samples was calculated as shown in Figure 1b. With the increase in MnO content, the relative intensity of Bi_2_O_3_ and Zn_2.33_Sb_0.67_O_4_ were both enhanced, which indicated that MnO content influenced the content of the intergranular phase.

To further study the effects of MnO doping on the microstructure of ZnO varistor ceramics, EDS was conducted along the yellow lines in Figure 1c–f. Element distribution was measured traversing several ZnO grains, grain boundaries and intergranular phases. It can be observed that Zn was mainly distributed in the grains (shown in black in the figure), Bi existed in light-colored intergranular phases, while Sb mainly existed in the spinel phase. With the increase in MnO content, the peak of Mn was gradually close to that of Sb, which showed that most of the Mn existed in the intergranular phase and a small portion of it was dissolved in the grain.

**Figure 2:**
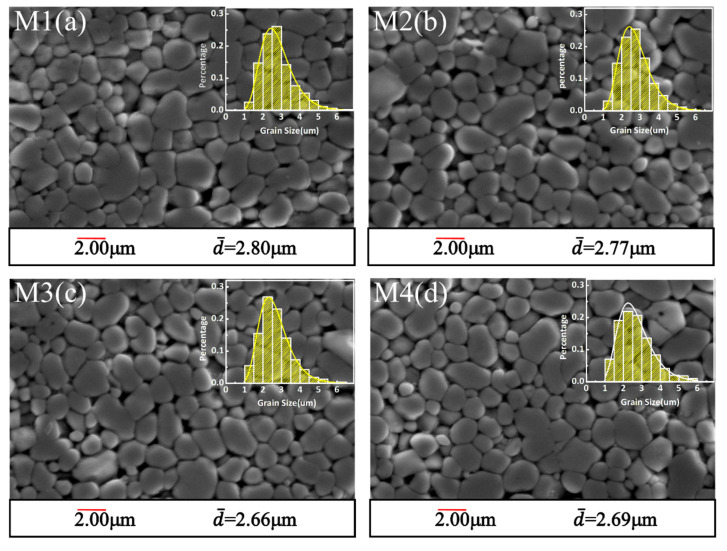

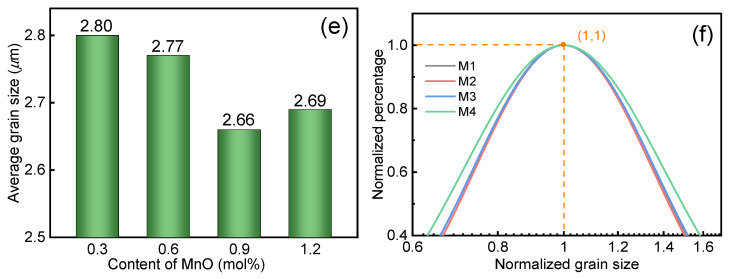
(**a**–**d**) SEM pictures, (**e**) average grain size and (**f**) normalized probability plots of the ZnO varistor ceramics.

Figure 2a–d display the SEM pictures of the samples. The grains, grain boundaries and intergranular phases were clearly observed. The grain size distribution was relatively uniform with a small amount of pores. The relative densities of the samples (*ρ*) are measured and listed in Table 1 and show a good densification [13]. Because of the usage of the nano-sized ZnO powders, the average grain sizes (*d*) of the samples were about 2.7 μm, which were much smaller than those of traditional ZnO varistor ceramics prepared from micro powders [34]. With the increase in MnO content, the intergranular phase inhibited the growth of grains, so that the average grain size shown in Figure 2e presented a decreasing trend.

In addition, the uniformity of grain size distribution (GSD) could not be ignored while reducing the average grain size. The GSDs of all the samples were statistically measured, which followed the lognormal distribution, as shown in the insets of Figure 2a–d. The normalized curves of the probability density function were compared, as shown in Figure 2f, and the coefficients of nonuniformity of GSD (*ε*) were calculated according to the following formula [5]:

(1) $$E(X)=e^{\mu +\sigma^{2}/2},$$

(2) $$D(X)=\sqrt{e^{\sigma^{2}}-1}(e^{\mu +\sigma^{2}/2}),$$

(3) $$\varepsilon =\frac{D(X)}{E(X)}=\sqrt{e^{\sigma^{2}}-1},$$

where *μ* is the logarithmic mean, *σ* is the logarithmic standard deviation, *E*(*X*) is the mean value of the lognormal distribution, *D*(*X*) is the standard deviation of the lognormal distribution, and *ε* is the coefficients of non-uniformity. The coefficients of nonuniformity are listed in Table 1. Combined with the half peak width of probability density function curves and non-uniformity coefficient, it can be seen that the GSD of sample M4 is the most non-uniform. This indicates that the excessive intergranular phase in sample M4 makes the grain non-uniform by the pinning effect.

**Figure 3:**
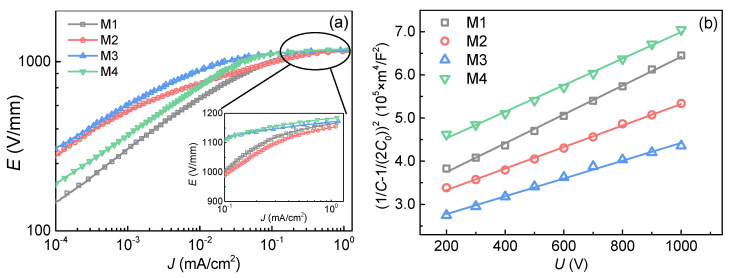
(**a**) Nonlinear *J-E* curves and (**b**) (1/*C* − 1/(2*C*_0_))^2^~*U* curves of ZnO varistor ceramics.

The nonlinear *J*-*E* characteristics of the ZnO varistor ceramics are shown in Figure 3a, from which the potential gradient (*E*_1mA_), nonlinear coefficient (*α*), and the leakage current density (*J*_L_) were calculated [35] and shown in Table 1. It is observed that the breakdown fields of all the samples were higher than 1000 V/mm due to the rather small grain sizes of 2.7 μm. The nonlinear coefficients of samples M3 were improved to 47.9 and the leakage current decreased to 8.4 μA/cm^2^ by doping 0.9 mol% MnO.

Moreover, it is well known that the nonlinear coefficient is relevant to the Schottky barrier height at the grain boundary [1]. Therefore, capacitance–voltage (*C*-*V*) characteristics were used to calculate the parameters of Schottky barrier [36,37]:

(4) $${\left(\frac{1}{C}-\frac{1}{2C_{0}}\right)}^{2}=\frac{2(\phi_{B}+U_{gb})}{e\varepsilon_{0}\varepsilon_{r}N_{D}},$$

where *C* is the barrier capacitance at bias voltage, *C*_0_ is the barrier capacitance with no bias voltage, *ϕ*_B_ is the barrier height, *N_D_* is the donor density, *U_gb_* is DC bias at the single crystal interface, and *ε_r_* and *ε*_0_ are the permittivity of ZnO and vacuum, respectively. Consequently, (1/*C* − 1/(2*C*_0_))^2^~*U* curves are plotted in Figure 3b, which shows a good linearity. The barrier height(*ϕ*_B_), density of the donor (*N_D_*), density of the interface state (*N*s) and width of the depletion layer(*t*) were calculated by (1/*C*−1/(2*C*_0_))^2^~*U* curve, as shown in Table 1.

It can be observed that the high barrier height of sample M3 was the main reason for its great nonlinearity. Furthermore, the density of the donor and interface state of sample M3 were both larger than those of other samples. It is well known that the Mn^2+^ dissolved in grains mainly replaced the lattice position of Zn^2+^ because of the similar radius. Therefore, it had little influence on the electron carrier concentration [31]. However, most of the Mn element was found to segregate at the grain boundary, which was reported to increase the concentration of V″_Zn_ and V′_Zn_ [38]. The depletion layer should satisfy the following the principle of electric neutrality [38]:

(5) $$n+[{V^{\prime }}_{Zn}]+2[{V^{\prime \prime }}_{Zn}]=p+[Zn_{i}^{\cdot }]+2[Zn_{i}^{\cdot \cdot }]+[V_{o}^{\cdot }]+2[V_{o}^{\cdot \cdot }],$$

where *n* is the electron carrier concentration and *p* is the hole carrier concentration. In order to satisfy the principle of electric neutrality, *N*_D_ increased. *N*_D_ and *N*_s_ satisfy the following relationship with *ϕ*_B_ [1]:

(6) $$\phi_{B}=\frac{e^{2}N_{S}^{2}}{2\varepsilon_{0}\varepsilon_{r}N_{D}}.$$

The interface state density of sample M3 increased, leading to the growth of the barrier height and nonlinear coefficient [39]. However, when MnO was over doped in sample M4, the electrical properties became worse due to the excessive intergranular phase. Therefore, combined with the microstructure, electrical parameters and barrier height, it can be concluded that sample M3 has the best performance when doping 0.9 mol% MnO.

## 4. Conclusions

In this study, ZnO varistor ceramics with a high performance were prepared. The increased potential gradient of 1172 V/mm was mainly attributed to the usage of nano powders, with the average grain size reduced to only 2.6 μm. The addition of MnO helped to increase the nonlinear coefficient to 47.96 and lower the leakage current to 8.4 μA/cm^2^. When doping a moderate amount of MnO, most of the Mn element, which segregated at grain boundary, enhanced the density of interface states, resulting in a high Schottky barrier height.

## Figures and Tables

**Table 1:** Parameters of ZnO varistor ceramics.

Samples	*d* (µm)	*ε*	*ρ*	*E*_1mA_ (V/mm)	*α*	*J*_L_ (μA/cm^2^)	*ϕ*_B_ (eV)	*N*_D_ (10^22^ m^−3^)	*N*s (10^16^ cm^−2^)	*t* (nm)
M1	2.80	0.314	97.1%	1163	15.25	40.1	1.42	7.87	6.28	399
M2	2.77	0.308	97.3%	1151	15.28	30.7	1.43	8.41	6.54	388
M3	2.66	0.316	97.6%	1172	47.86	8.4	1.69	12.59	8.68	345
M4	2.69	0.350	97.4%	1179	35.14	22.4	1.53	6.72	6.03	449

## Data Availability

Not applicable.
